# Real-time location systems technology in the care of older adults with cognitive impairment living in residential care: A scoping review

**DOI:** 10.3389/fpsyt.2022.1038008

**Published:** 2022-11-10

**Authors:** Lynn Haslam-Larmer, Leia Shum, Charlene H. Chu, Kathy McGilton, Caitlin McArthur, Alastair J. Flint, Shehroz Khan, Andrea Iaboni

**Affiliations:** ^1^KITE Research Institute, Toronto Rehabilitation Institute, University Health Network, Toronto, ON, Canada; ^2^Lawrence S. Bloomberg Faculty of Nursing, University of Toronto, Toronto, ON, Canada; ^3^School of Physiotherapy, Dalhousie University, Halifax, NS, Canada; ^4^Department of Psychiatry, Temerty Faculty of Medicine, University of Toronto, Toronto, ON, Canada; ^5^Centre for Mental Health, University Health Network, Toronto, ON, Canada

**Keywords:** RTLS (Real-time location system), cognitive impairment, dementia, older adults, residential care, long-term care, remote monitoring, wearable technological device

## Abstract

**Introduction:**

There has been growing interest in using real-time location systems (RTLS) in residential care settings. This technology has clinical applications for locating residents within a care unit and as a nurse call system, and can also be used to gather information about movement, location, and activity over time. RTLS thus provides health data to track markers of health and wellbeing and augment healthcare decisions. To date, no reviews have examined the potential use of RTLS data in caring for older adults with cognitive impairment living in a residential care setting.

**Objective:**

This scoping review aims to explore the use of data from real-time locating systems (RTLS) technology to inform clinical measures and augment healthcare decision-making in the care of older adults with cognitive impairment who live in residential care settings.

**Methods:**

Embase (Ovid), CINAHL (EBSCO), APA PsycINFO (Ovid) and IEEE Xplore databases were searched for published English-language articles that reported the results of studies that investigated RTLS technologies in persons aged 50 years or older with cognitive impairment who were living in a residential care setting. Included studies were summarized, compared and synthesized according to the study outcomes.

**Results:**

A total of 27 studies were included. RTLS data were used to assess activity levels, characterization of wandering, cognition, social interaction, and to monitor a resident’s health and wellbeing. These RTLS-based measures were not consistently validated against clinical measurements or clinically important outcomes, and no studies have examined their effectiveness or impact on decision-making.

**Conclusion:**

This scoping review describes how data from RTLS technology has been used to support clinical care of older adults with dementia. Research efforts have progressed from using the data to track activity levels to, most recently, using the data to inform clinical decision-making and as a predictor of delirium. Future studies are needed to validate RTLS-based health indices and examine how these indices can be used to inform decision-making.

## Introduction

As those with dementia require increasing support for daily functioning, many relocate to assisted living environments. The umbrella term “residential care” is often used to reflect the continuum of assisted living environments designed to facilitate and support an older adult’s functional independence. The majority of those living within a residential care setting in industrialized countries have dementia or cognitive impairment (1–3). As most of these residential care settings have a limited staff-to-resident ratio, technology utilization may potentially help to alleviate resource gaps and augment care delivery.

There have been several reviews on the use of technologies in older adults with dementia to measure clinical variables, such as detection of behavioral symptoms (4), monitoring of treatment response (5), prediction of falls risk (6), gait analysis (7), and physical activity levels (8). These reviews were not exclusive to the uses of technology in residential care settings, and they examined various wearable and non-wearable and environmental sensors. A systematic review by Lynn et al. (9) identified technologies used in residential care settings. The technology categories included were largely *resident-facing* technologies, such as telecare, light therapy, robotics (e.g., robotic companion), wellbeing and leisure (e.g., touch screen devices, watches to measure sleep cycles), simulated presence and orientation (e.g., audio/video recordings), and activities of daily living (e.g., handwashing, taking medication memory aids). We are unaware of any reviews examining *provider-facing* technologies designed to support clinical decision-making in residential care.

One particular provider-facing technology in residential care are real-time locating systems (RTLS). RTLS, also known as indoor positioning or location systems, are primarily used for tracking individuals and equipment in indoor environments in real or near-real time or incorporated into nurse call or safety systems (10, 11). A RTLS typically consists of a wearable device that contains a sensor (e.g., a tag or bracelet) that is worn by an individual, a number of environmentally embedded receiver devices (e.g., beacons on the ceiling), and software in order to visualize location data on a facility map, connected on a wireless network to continuously track people or items in real time. Non-wearable sensor technologies can be deployed as RTLS in clinical settings, such as near-field radio-frequency ID (RFID) tags or passive infra-red (IR) sensors, which detect movements of individuals passing through doors or at a room-level scale. Higher accuracy, wearable systems such as Bluetooth or Ultra-wide band (UWB) are used when the intention is to collect within-room movement patterns at a regular sampling rate over time. RTLS provides a vast amount of data on an individual’s location over time and can characterize movement through a well-defined target environment. RTLS installations have been studied in a wide variety of health care settings to monitor individuals’ movements (e.g., residents/patients, staff) and assets (e.g., surgical equipment) (12–15).

In a previous technical review, the authors identified the ways in which meaning can be extracted from RTLS data to describe human behaviors in a variety of different settings (healthcare, education, workplaces, shopping malls, art galleries) (11). We have also completed a previous systematic review to identify factors that affect the implementation of RTLS for use with persons living with dementia in long-term care homes (16). However, neither of these reviews addressed the ways in which RTLS data was being used as a clinical outcome or how the data was used to inform clinical decision-making processes. As this type of technology becomes more common within residential care settings, there is a need to understand the potential uses of the collected RTLS data to provide clinical insights and augment healthcare decisions.

Thus, this scoping review aims to describe the available evidence on the use of data from RTLS technology to inform clinical measures, demonstrate validation of clinical measurements, or augment healthcare decision-making in persons with cognitive impairment living in residential care. A preliminary search of MEDLINE, the Cochrane Database of Systematic Reviews, and *JBI Evidence Synthesis* did not identify any current or in-progress scoping reviews or systematic reviews on this topic. Understanding this literature is important, as synthesizing this information can shape future studies to identify the optimal use of RTLS technology in this population of older adults.

## Review questions

1. How are data from RTLS technologies used to support the clinical care of older adults with cognitive impairment residing in a residential care setting?

2. How have RTLS data been used to inform or validate clinical measures for the clinical care of people with cognitive impairment in residential care settings?

## Inclusion criteria

### Participants

This scoping review considered studies of persons 50 years of age or older who lived in a residential care setting. We opted to use an age criterion to exclude residential care settings for people with cognitive impairments that do not primarily care for older adults, such as group homes. A cut-off of 50 years old was selected to avoid excluding individuals with early onset dementia. Studies were included if the majority of study participants had cognitive impairment or dementia.

### Concept

This review considered studies that examined the use of RTLS. RTLS consists of a software application and reference points that detect and synthesize positioning data from wireless transmitters worn by people or attached to objects, enabling the collection of several indices (e.g., tracking movement and activity levels) that locate a person in space over time. The technology may include RFID, UWB, GPS, or other sensor-based systems. Outcomes of interest for this review included identifying how clinicians use this RTLS data to augment clinical assessment and clinical decision-making processes.

### Context

This review considered studies in which study participants resided within an assisted living environment, a residential environment that supports or enables older adults with or without cognitive impairment. For this review, a residential care environment includes assisted living facilities (e.g., retirement homes or group homes), long-term care facilities (e.g., nursing homes), or special dementia care units within the community or hospital settings.

### Types of sources

For this scoping review, we included published original research exploring the use of RTLS technology in older adults with dementia living in a residential care setting. We considered quantitative, qualitative, case studies, case reports, and mixed methods study designs for inclusion. Commentaries and publications with hypothetical uses of the technology were excluded (Table 1). Conference papers and abstracts were included, but if they did not contain clinical data, or were duplicated in a peer-reviewed publication, they were excluded. We did not include books, study protocols, or previous reviews.

**TABLE 1:** Inclusion and exclusion criteria.

Inclusion criteria	Exclusion criteria
***Participants*** ○ Adults older than 50 years of age ○ Majority of study participants with cognitive impairment or dementia ***Concept*** ○ Use of real-time locating systems (may include GPS or other sensor-based systems) ***Context*** ○ Study participants resided within an assisted living environment	1. Studies that discuss implementation issues or ethical issues (not reflective of direct clinical care), 2. Studies that only describe the potential benefits of the technology for this population, 3. Study abstracts or conference posters with no data, or that are represented by a later publication 4. Study protocols, or articles only describing technology set-up 5. Studies that do not use the technology of interest (tracking a person’s location over time) (e.g., excludes activity monitors, accelerometers, or bed-exit sensors) 6. Use of “WanderGuard (R)” type of technology that solely prevents exit but does not otherwise track location 7. Where the focus of the technology is serious gaming 8. Environmental mapping technology 9. Review papers

The review was limited to studies published in English. A systematic review has demonstrated that restricting the search strategy to English-language publications has little impact on the conclusions for most medical topics (17).

## Materials and methods

This scoping review was conducted in accordance with the JBI methodology for scoping reviews (18) and the Preferred Reporting Items for Systematic reviews and Meta-Analyses extension for Scoping Reviews (PRISMA-ScR) Statement (19) (Supplementary Appendix 3). The PRISMA-ScR framework provides guidance on the reporting of items required within a scoping review. The protocol for this scoping review was published *a priori* via the Open Science Framework.^1^ The protocol was amended to modify the exclusion criteria after our initial search: for example, we omitted papers that “mapped” clinical environments, but patient data was not collected.

### Search strategy

The search strategy aimed to locate peer-reviewed published studies. This scoping review follows the work of two previous reviews (11, 16) that focused on implementation/ethics of RTLS technologies and data analytic approaches for RTLS data. We worked with an information specialist who is trained in comprehensive searching methods for knowledge synthesis and has experience in conducting scoping reviews to plan and execute the search strategy. The text words contained in the titles and abstracts of relevant articles, and the index terms used to describe the articles were used to develop a full search strategy. The search strategy, including all identified keywords and index terms, was adapted for each included information source (Table 2). The search encompassed literature from the inception of each database to September 2021. Reference lists of the included literature were hand searched for additional relevant studies. A sample of the search strategy is provided in Supplementary Appendix 1.

**TABLE 2:** Search strategy terms.

Concept	Keywords
Cognitive impairment	Cognition, memory, memory loss, Alzheimer/Alzheimer’s disease, dementia, neurocognitive disorder, cognitive impairment/dysfunction/disorder, lewy body
Residential care	Long term care, long-term care, LTC, nursing home(s), old age home, home for the aged, retirement home(s), retirement community, residential care, residential facility(ies), nursing facility(ies), group home(s), assisted living, convalescent care, long stay/longterm, old age home, old age facility
RTLS	Real time monitor, real time location/systems, indoor positioning, indoor locating, continuous surveillance, motion tracking, tracking device, location monitoring, monitoring system, technology, GPS, RTLS, radio-frequency ident/RFID, Ultra-wide band/UWB, Bluetooth

The databases searched include: Embase Classic + Embase (Ovid) Medline (including Epub Ahead of Print, In-Process and Other Non-Indexed Citations, Ovid MEDLINE^®^ Daily and Ovid MEDLINE^®^, in Ovid), CINAHL (EBSCO), APA PsycINFO (Ovid) and IEEE Xplore. Unpublished studies, conference abstracts, and gray literature were not included.

### Source of evidence selection

Following the search, all citations were uploaded to Covidence systemic review software (Veritas Health Innovation, Melbourne, Australia) and duplicates were removed. After a pilot test of screening criteria, titles and abstracts were screened by two independent reviewers (LH-L, LS) for assessment against the inclusion criteria for the review. Potentially relevant papers were retrieved in full and uploaded into Covidence. Two reviewers assessed these studies independently to determine if they met the study criteria (LH-L, LS). Any disagreements between the reviewers at each stage were resolved by consensus or with a third reviewer (AI). For each study a quality appraisal was conducted using the appropriate JBI Quality Appraisal tool (Supplementary Appendix 2).

### Data extraction

Data were extracted from papers included in the scoping review by two independent reviewers (LH-L, LS) using a data extraction tool within Covidence and developed by the reviewers. The data extracted included specific details about the aim of the study, study design, participant characteristics, years data was collected (if available), associations/relationships to other studies, residential home characteristics, the type of tracking technology, how the technology was used, measurements used, comparisons between measurements, and key findings relevant to the review question. Any disagreements that arose between the reviewers were resolved through discussion or with a third reviewer. Authors of papers were contacted to request missing or additional data, where required. Upon review of the final data extraction sheet, we used a content analysis approach to identify common themes amongst the literature. We were then able to compare and contrast findings in relationship to these themes.

### Data analysis and presentation

Results are reported graphically with tables when possible. The narrative that accompanies the tables further describes the body of literature. The review findings are reported in five broad themes: levels of activity, wandering or risk of wandering, cognitive status, proximity to others/social interaction, and other measures of health status. These themes were identified as potential areas of clinical care supported by RTLS technology through the process of the review.

## Results

### Study inclusion

**FIGURE 1:**
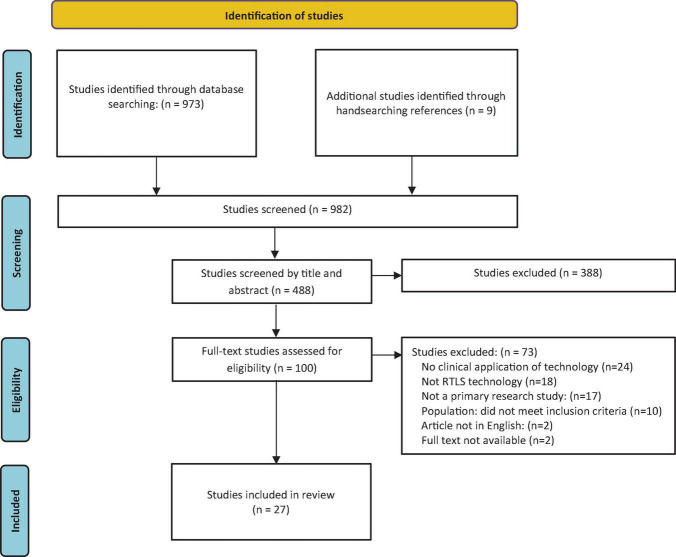
Search results and study selection and inclusion process (19).

A total of 982 articles were identified and uploaded to Covidence for screening. Of these, 492 were duplicates. At the title and abstract phase, 488 studies were screened, with 388 studies found ineligible. There were 100 full-text studies assessed for eligibility through full-text screening, and 73 were excluded (see Figure 1). Examining the included papers’ reference lists resulted in nine additional studies for inclusion.

Reasons for exclusion were as follows: no clinical application of technology (20), not RTLS technology (18), not a primary research study (17), the population did not meet inclusion criteria (10), article not in English (2), and no full text available (2). The resulting 27 studies were included in the review.

### Characteristics of included studies

The majority of the papers were from the United States (*n* = 12) (12, 13, 20–29), and Japan (*n* = 9) (30–38), and one each from Italy (39), Germany (14), France (40), Austria (41), Netherlands (42), and Hong Kong (43). No studies were excluded based on age criteria. The youngest participant across all studies was 54.

Studies were published across the date range of 2002 to 2021. Only one study was published in 2002 (40) with remaining studies published after 2007. The included research studies used various research designs, including cross-sectional (*n* = 16) (14, 20, 23–25, 28, 30–34, 36–38, 41, 43), longitudinal (*n* = 5) (12, 13, 22, 27, 42), case reports (*n* = 4) (26, 35, 39, 40), and one of a case series (21).

Ten studies used UWB technology (12, 13, 20, 22–25, 27, 28, 41). Other technologies used included RFID (IC tag) (*n* = 8) (30, 31, 33–38), radio waves (Emerald ^®^) (*n* = 3) (21, 26, 29), Bluetooth (*n* = 3) (32, 39, 43), and one each of GPS technology (42) passive IR (40) and an unspecified wireless mesh network (Wi-Fi) (14).

The quality of the evidence is reported in Supplementary Appendix 2. Study quality ranged from poor (21, 25, 26, 32, 38, 43), moderate (12, 13, 20, 23, 24, 28–31, 34, 36, 37, 39–41, 44), to good (14, 22, 27, 42). Strengths across the studies included in-detail descriptions of the RTLS implementation in the residential setting. Common limitations were unclear recruitment processes, and not all studies documented how consent was obtained.

### Review findings

The results of this scoping review are discussed under the following themes: levels of activity, characterization of wandering, cognition, social engagement, RTLS used to monitor health status or effect of an intervention, and other outcomes (Table 3). The table has been organized from publication date, highlighting how the focus of RTLS use in this population has changed over time.

**TABLE 3:** Themes of RTLS use in older adults with dementia in residential living (by publication dates).

	Author	Sample size, period of monitoring	Age	Themes					
				Levels of activity	Characteriz ation of wandering	Cognitive status	Social interaction	Monitoring health status or effect of an intervention	Other study comparisons
1.	Chan et al. (40)	*n* = 1 39 days	92	X					Night activity compared to nursing documentation
2.	Greiner et al. (33) study period 2005	*n* = 13 1 week	Mean 69.6 ± 7.5	X	X				Movement patterns compared to direct observation (accuracy)
3.	Miyoshi et al. (36) study period 2006–2007	*n* = 23 Mean 59 days	Mean 69.6 ± 10.8	X					Activity level to change in body weight
4.	Makimoto et al. (34) study period 2006	*n* = 8 12 days	Mean 76 ± 5.3	X					Temporal patterns of ambulation over time
5.	Nakaoka et al. (37) study period 2006–2007	*n* = 23 Mean 53 days	Mean 70 ± 10.6		X				Distances paced/lapped as a proportion of distance moved
6.	Yamakawa et al. (35) study period 2006–2007	*n* = 1 4 weeks	62					Environmental control intervention	
7.	Yayama et al. (30) study period 2006–2007	*n* = 30 Median 7 days	Mean 67.6 ± 13.1	X	X				Wandering compared to nursing documentation
8.	Yamakawa et al. (31) study period 2008–2009	*n* = 35 Mean 69 days	Mean 74.4 ± 8.3	X					Night activity compared to nursing documentation
9.	Liao et al. (38) study period 2008–2009	*n* = 7 Mean 69 days	Range 71–93					Changes in activity patterns after benzodiazepine	
10.	Kearns et al. (24)	*n* = 14 30 days	Range 63–92			X			Association of tortuosity to cognitive impairment
11.	Kearns et al. (20)	*n* = 25 30 days	Range 59–92		X	X			Correlations between speed and cognitive impairment
12.	Grunerbl et al. (41)	*n* = 6 14 days	Mean 88.6	X			X	Recognition of resident states/trends	
13.	Kearns et al. (23)	*n* = 49 1 year	76.9 ± 11.9					Ability to predict falls	
14.	Bowen et al. (28)	*n* = 8 3 weeks	Not reported*				X		Locations and social interaction of residents positive for MRSA/VRE
15.	te Boekhorst et al. (42)	*n* = 8 (GPS) 3 × 2 months	83 ± 9.2					Quality of life measurement compared to restraint use	
16.	Bowen and Rowe (13)	*n* = 26 Mean 16 weeks	Mean 79					Ability to predict falls	
17.	Kumar et al. (25)	*n* = 10 1 year	76.9 ± 11.9			X			Trajectory and cognitive impairment
18.	Jansen et al. (14)	*n* = 65 2 days	Mean 82.9 ± 9.6	X		X			Gait speed, apathy, depressive symptoms related to life space
19.	Vahia et al. (26) study period 2017	*n* = 1 70 days	85	X	X				Periods of agitation (wandering or pacing) compared to documentation
20.	Bowen et al. (12)	*n* = 26 Mean 14 weeks	Mean 79.0 ± 8.9 (58–94)			X			Influence of cognitive impairment on speed and distance
21.	Okada et al. (32)	*n* = 19 3 months	Mean 84.56 ± 5.25	X					Activity level and mobile robot interactions
22.	Bowen and Rowe (27)	*n* = 22 8 months	Mean 79.0 ± 8.4		X				Wandering and ADL function
23.	Yang et al. (43)	*n* = 50 12 weeks	Not reported	X			X		Mobility and room accommodations
24.	Kearns et al. (39) study period 2020	*n* = 64 1 year	Not reported				X		Develop popularity index/relational index
25.	Bowen and Cacchione (22)	*n* = 23 18 months	Mean 79.9					Prediction of delirium (falls/infection)	
26.	Zhang et al. (29)	*n* = 3 3 months	Range 73–88	X				Behavioral trajectories of COVID-19 recovery	
27.	Bowen and Rowe (21)	*n* = 2 115 and 79 days	80, 85	X				Use technology to inform medication changes	

*Author confirmed residents were 55 and older Level of activity.

### Compared to nursing documentation

In the earliest study in 2002, investigating the use of RTLS technology as a clinical tool, Chan et al. (40) validated the outputs of infra-red motion sensors against nursing documentation of a single patient across 39 nights. They identified that the nursing staff underestimated the patient’s nightly travel patterns, and the location data provided a more accurate assessment of resident activity. The ability of technology to provide a more precise description of a resident’s activity was supported by the work from Osaka, Japan (30, 31, 33). In this series of studies, the authors compared resident movement data to nursing observations, finding little agreement between location data and staff documentation.

### Patterns of daily activity

Several studies have examined patterns of movement through the day, such as time spent outside of the bedroom or in shared spaces. For example, Bowen and Rowe (13) described the level of activity and activity variance between residents, reporting a high association between time spent away from room and institutionally scheduled routines, such as mealtimes. Residents with better walking abilities were observed to stay in common areas for extended periods and changed life-space areas less frequently. Those with inferior walking ability remained more often in their rooms. Grunerbl et al. (41) were also able to identify the daily activity habits of each participant, although they did not match these activities to walking ability. Grunerbl et al. (43) differentiated mobility patterns into social spaces according to the environment’s design. They determined that residents with rooms that open directly into social spaces had higher social withdrawal tendencies. Rooms farthest from social areas and those with no direct visibility to social areas were also associated with social withdrawal.

Other studies described daily activity patterns as they relate to an underlying diagnosis. Makimoto et al. (34) described the temporal activity patterns of several residents within a special dementia unit. They found that those with Alzheimer’s dementia had longer distances walked (mean 575 meters) compared to those with vascular dementia (mean 312 meters). Zhang et al. (29) tracked the activity of three residents as they recovered from COVID-19. They were able to demonstrate that sleep and motor abnormalities persisted in these residents for months after recovery compared to the baseline data which was obtained at the start of monitoring. For the first patient, the monitoring began on the day they returned from hospital (Day 11 after testing positive), the second patient’s monitoring began on Day 6, and the third on Day 0.

#### Characterization of wandering

Several studies of wandering have correlated location data to assessment tools used by nursing staff. Yayama et al. (30) identified that of the 23 Japanese Algase Wandering Scale (J-AWS) items, eight could be compared to movements measured by the monitoring technology. The temporal movements were compared to the first five items on the J-AWS, namely, the degree of restlessness throughout meal times, and spatial movements were compared to the pacing measurements. Residents rated as “wanderers” by staff had a longer distance walked per day than those rated as “non-wanderers.” The authors highlighted that staff assessment alone was likely insufficient to evaluate wandering behavior. They also found a poor correlation between night-time activity and staff J-AWS evaluation, although wandering through the night was a common occurrence. Bowen and Rowe (27) compared RTLS data to activities of daily living (ADL), measured by the Barthel index and the Functional Independence Measure, to assess the positive or negative effects of wandering on the ability to perform ADLs. This study found that an increased number of wandering episodes per week are associated with increased ability to perform ADLs. In contrast, an increased wandering distance was associated with a reduced ability to perform ADLs. Although Nakaoka et al. (37) did not compare data to a wandering tool, they used technology to characterize a resident’s pacing and lapping activities as a proportion of distance moved. They found the median number of pacing movements per day ranged from 2 to 52, with a high correlation to the median distance per day.

Two studies reported detailed mobility of one resident over time in an attempt to characterize wandering patterns. Greiner et al. (33) compared the movements of one resident’s wandering to observational records, finding that there was no pattern to his wandering, and caregivers had differing interpretations of the meaning of his wandering behaviors. Vahia et al. (26) compared location data on one resident over 70 days to nursing staff documentation. The data allowed them to identify patterns in levels of agitation as evidenced by night-time wandering and excessive pacing in the days following a family visit. Again, they found little agreement between nursing documentation and location data.

#### Association of mobility to levels of cognition

Three studies (20, 24, 25) link patterns in mobility to degree of cognitive impairment. They extracted various measures from location data over time (e.g., speed, path tortuosity) and examined the association between these features and cognitive impairment or dementia severity. A greater tortuosity (i.e., quantization of trajectory of a path with several turns) of the walking path predicted a higher level of cognitive impairment (20, 24, 25). Similarly, Bowen et al. (12) demonstrated that sustained gait speed was lower in those with severe cognitive impairment, and cognitive impairment was associated with longer but slower sustained walking.

Jansen et al. (14) used activity levels to determine that lower cognitive function is associated with more time away from their room. They hypothesized that those with lower levels of cognitive function and subsequent inability to independently way-find are often bound to stay in public areas longer.

#### Social interaction

There has been growing interest in using RTLS technology to measure degree of social interaction between nursing home residents. Grunerbl et al. (43) reported that the location data of those in a three-bedroom accommodation had the highest probability of being in a social space, while residents in a five-bedroom accommodation had a higher probability of being in a co-resident’s room. In a similar effort to quantify relationships between residents, Bellini et al. (39) developed the Relational Index and Popularity Index to quantify clusters of residents and close friendships within the community. They were able to demonstrate an objective change in the sociability pattern within the community during COVID-19 pandemic (Jan 2020-June 2020), where residents experienced longer times in isolation during the pandemic. Similarly, Okada et al. (32) used resident activity levels to calculate time spent in one’s room vs. shared spaces, on the assumption that residents who stay in the shared space or in other’s rooms are more socially active.

In addition to social interaction, Bowen et al. (28) demonstrated a unique clinical applicability of collecting social interaction data in the domain of infection prevention and control. This study reviewed movements of residents who were methicillin-resistant *Staphylococcus aureus* (MRSA) and vancomycin-resistant *Enterococci* (VRE) positive, and were able to show that residents MRSA and VRE positive residents interacted with other residents (both positive and negative) regularly within the communal setting.

In terms of detecting negative interactions with co-residents, the study by Grunerbl et al. (41) identified episodes of aggressive behavior in one resident when they were near another resident. Other than describing the proximity and time together the residents, there are no studies examining the quality (e.g., resident behaviors signifying level of interest) of the social interactions.

#### Real-time location systems to monitor health status or effect of intervention

Multiple studies demonstrated how RTLS technology can be used to detect changes in a resident’s health status. Miyoshi et al. (36) explored the relationship between bodyweight and levels of activity. They were able to demonstrate weight gain in those with higher sedentary times, and weight loss in one resident who walked over 5 kilometers per day. The study by Grunerbl et al. (41) used UWB technology data to correlate location and a resident’s change in “broad classes of wellbeing” (positive or negative). A resident’s state of wellbeing was measured by the duration of stay in one area, the transition between areas, the ratio of the duration of stay and changes, and distribution over an area. They demonstrated that the technology could recognize a resident’s positive or negative state of wellbeing with improving accuracy over a more extended period (14 days). If a resident’s state of wellbeing fluctuated over the course of a day the prediction by the technology was not as accurate.

With UWB technology, Kearns et al. (23) demonstrated that a resident’s increased path tortuosity is associated with an increased risk of falling. Bowen and Rowe (13) also identified that walking patterns are associated with higher fall risk. Specifically, they identified residents who spend more time walking are at increased risk of falls. Bowen and Cacchione (22) used RTLS across two nursing homes and 23 residents for 18 months to examine whether mobility changes can be indicative of changes in health status. When reviewing average weekly motor behavior of the larger sample, residents with delirium walked farther (543 meters) and longer (35 min). Additionally, the authors identified that residents with an increased gait speed and continuous monthly decreases in physical and cognitive performance were most likely to experience a fall. Most recently Zhang et al. (29) used information derived from location data to describe changes in behavior (e.g., increased gait speed, increased activity levels) that were associated with recovery from COVID-19. They were also able to identify that one resident had worsening mobility and changes to sleep which may have been secondary to either experiencing COVID-19, or as a result of prolonged periods of isolation.

RTLS technology has also been used to monitor changes associated with medication administration. Liao et al. (38) used location data to provide descriptions of activity-based changes after a dose of brotizolam, notably a paradoxical increase in night-time wandering and daytime restlessness. In a case series, Au-Yeung et al. (21) demonstrated how passive IR technology can be used to detect changes in a resident’s behavior, leading to successful changes in medication treatments. In one scenario, the technology was used to identify that increasing sedentary times as a marker of decline in cognitive status. In a second case, the technology detected that a resident experienced an unusual motion signal during sleeping hours, which was identified as risperidone-induced periodic limb movements. They reduced the risperidone dose, leading to a resolution of the periodic limb movements.

In a case study, Okada et al. (32) used Integrated Circuit (IC) tag technology to demonstrate the impact of two interventions on the sleep-wake cycle. In the first intervention, they closed all doors within the unit, thereby increasing the resident’s daytime activity levels (decrease in the number of episodes of entering and napping in other resident rooms), which resulted in a significant decrease in night-time movement. The second intervention involved nurse-led mobility sessions, in which the resident resisted participation in the mobility sessions, contributing to increased agitation and night-time movements.

Finally, te Boekhorst et al. (42) implemented a GPS-based system as a restraint reduction intervention in residents at risk of elopement or falls. There was no significant effect of the system on the use of restraints (with a small number of restraints used overall), and no impact on resident quality of life.

## Discussion

This scoping review has described how data from RTLS technologies have been used to support the clinical care of older adults with cognitive impairment in a residential care setting. While we also sought to identify validated RTLS-based clinical measures, we found few studies that have attempted this validation.

Most clinical use cases for this technology have focused on describing mobility or activity levels. Early studies compared RTLS data to nursing documentation and highlighted that these comparisons were difficult. An attempt was made to validate clinical wandering scales (e.g., RAWS-CV, J-AWS), but results were not promising secondary to low correlation between RTLS data and staff documentation. Residents are not constantly observed, and healthcare staff are not trained to detect changes in a resident’s gait, speed, or path. Thus, subjective staff assessments of a resident’s walking activity/wandering diverge substantially from the technology’s objective measure. Many patients with dementia may experience motor agitation (45), influencing patients to wander, and wandering is one of the most challenging care issues for people with dementia (46, 47). Given that clinician ratings so poorly capture the nature and extent of wandering, residents’ mobility and activity levels, it represents a promising target for RTLS technology.

Most recently, studies have focused on applying the data to describe social interaction for various purposes. From a systems perspective, data have been used to identify popular areas and traffic patterns within nursing homes, leading to the potential use of the data to aid in better nursing home design. There is a paucity of objective data that reports on the impact of environmental adaptations that may help to disguise exits or improve wayfinding within the unit. When examining resident interactions, authors have provided data on resident proximities, which was demonstrated to help trace communicable illnesses. However, there is little evidence available on the degree of interest, enjoyment or engagement of residents when they are in close proximity. There is potential for growth in this area, linking measurements of quality of resident interaction or social engagement to proximity data.

An under-explored area of research is the use of these technologies for evaluating residents’ psychological wellbeing. Jansen et al. (14) was the only study that specifically employed the Geriatric Depression Scale and Apathy Evaluation Scale. They identified that fewer depressive symptoms were significantly associated with more time away from a resident’s room. Cognitive impairment increases the risk for depressive symptoms (48, 49), and depression has been linked to increased sedentary time (50). Similarly, depression in nursing homes has been historically under-recognized and under-treated (51). Future studies that specifically examine the relationship between activity patterns and depression using similar scales would be helpful for clinicians. As an example, changes to level of activity may indicate a sign of depression. Pairing the technology findings with clinical assessments of mental health status may be beneficial to identify these potential relationships.

The body of literature reviewed in this study have provided multiple examples of the use of RTLS technology to aid in decision-making for resident care, mostly in case studies, with a dearth of clinical trials. However, some examples of objective data collection have immediate clinical applications. Miyoshi et al. (36) demonstrated that those who walk more are at greater risk of losing weight. Theirs was one of the first studies to objectively illustrate the impact of activity levels on weight change among residents with dementia. Clinicians may not easily estimate a resident’s energy requirements, as it is difficult to estimate the distances or levels of activity that residents are engaging in (52). Most recently, the case series by Peters et al. (18) and Kearns et al. (20) demonstrate how RTLS data can be used to direct medication treatments and better understand agitation triggers. With any case series studies, there is the limitation of generalizability of the data to other individuals and an over-interpretation of the data presented. However, the authors demonstrate a real-time clinical application of RTLS and how the data can subsequently be used to lead to optimal medication treatment.

There are several limitations within the body of work. Firstly, there is a lack of standardized assessments, which makes it difficult to compare results between papers (e.g., ADL and agitation scales differed between studies). None of the studies to date have successfully validated RTLS measures against a clinically important outcome or examined their diagnostic accuracy using receiver operating characteristic (ROC) curve analysis. As the quality of the evidence was overall poor to moderate, there were concerns related to sample size, inconsistent reporting of ethics, and descriptions of the datasets or the RTLS technology used. The duration of data collection varied widely. For example, Jansen et al. (14) collected data on 69 residents for only 2 days, whereas Bowen and Cacchione (22) collected data on 23 residents for 18 months. While there is some value in building evidence through case studies and observational studies, more extensive studies using population-level data in assisted living settings and advanced data science methods such as machine learning or deep learning, are needed.

Additionally, there are limitations to our scoping review process that are worth noting. First, scoping reviews have inherent limitations because the focus is to provide breadth rather than depth of information in a particular topic. This review provides a description of how RTLS technology is being used clinically in older adults with dementia living in residential care, and as such, the findings may not be applicable to other settings or populations. We also limited the review to studies in English, yet restricting reviews to English language publications appears to have little impact on conclusions of reviews (17).

### Implications for practice and future research

Research into and clinical use of RTLS technology has increased substantially over the last twenty years, while many important questions remain to address. Interpreting the distance, speed or pattern of a resident’s movements over time in relation to their health and wellbeing is not a straightforward task. In the current state, much remains to be learned about extracting clinically meaningful insights from RTLS data. There is also a need for clinical evaluative studies about whether these objective measures can be used to influence decision-making to effect better patient outcomes. Moving forward, it will be necessary for researchers to validate these location-based measures against clinically important outcomes and consider how they can be used within clinical decision-making algorithms.

## Author contributions

LH-L, LS, and AI performed the development of literature search protocol and contributed to text screening and data extraction. LH-L and LS performed initial analysis and compilation of results, and wrote the initial manuscript draft. CC, KM, CM, AF, SK, and AI performed review and editing. All authors have read and agreed to the published version of the manuscript.

## Footnotes

1
https://osf.io/3zny9/
