# Supplementary material for: Real-time location systems technology in the care of older adults with cognitive impairment living in residential care: A scoping review
Source: Front Psychiatry. 2022 Nov 10;13:1038008. doi: 10.3389/fpsyt.2022.1038008 (PMC9685159; doi:10.3389/fpsyt.2022.1038008)
Supplement: Supplementary file 2 [file Data_Sheet_2.docx]

**Appendix II: Quality Appraisals**

Cross-sectional studies

| Publication Year | Author | Were criteria for inclusion in the sample clearly defined? | Were the study subjects and the setting described in detail? | Was the exposure measured in a valid and reliable way? | Were objective, standard criteria used for measurement of the condition? | Were confounding factors identified? | Were strategies to deal with confounding factors stated? | Were the outcomes measured in a valid and reliable way? | Was appropriate statistical analysis used? | Rating | Rating comments |
| --- | --- | --- | --- | --- | --- | --- | --- | --- | --- | --- | --- |
| 2007 | Greiner | Yes | Yes | Yes | Yes | No | No | Yes | Yes | Moderate | Descriptive mobility data of one resident compared to observation |
| 2008 | Miyoshi | Yes | Yes | Yes | Yes | No | No | Yes | Yes | Moderate | Food intake, weight, mobility data |
| 2008 | Makimoto | Yes | Yes | Yes | Yes | Not applicable | Not applicable | Yes | Yes | Moderate | Descriptive of mobility data |
| 2010 | Nakaoka | Yes | Yes | Yes | Yes | Not applicable | Not applicable | Yes | Yes | Moderate | Descriptions of pacing/lapping |
| 2010 | Kearns | No | No | Yes | Yes | No | Unclear | Yes | Yes | Moderate | Limitation of poorly described setting |
| 2011 | Kearns | Yes | Yes | Yes | Yes | Yes | Unclear | Yes | Yes | Moderate | Calculations and procedure well described |
| 2011 | Grunerbl | Yes | Yes | Yes | Yes | Unclear | Unclear | Yes | Yes | Moderate | Some confusion about 'states' |
| 2012 | Liao | Yes | Yes | Yes | Yes | No | No | No | Yes | Poor | Measured distance moved post Rx administration (effect of other Rx?) |
| 2012 | Bowen | No | Yes | Yes | Yes | Not applicable | Not applicable | Yes | Yes | Moderate | Patients selected due to proximity to each other |
| 2012 | Kearns | Unclear | Yes | Yes | Yes | Not applicable | Not applicable | Yes | Yes | Moderate | Recruitment process unclear |
| 2012 | Yamakawa | Yes | Yes | Yes | Yes | No | No | Yes | Yes | Moderate | Methods well described |
| 2012 | te Boekhorst | Yes | Yes | Yes | Yes | Yes | Yes | Yes | Yes | Good | Few episodes of restraint use, QoL difficult measurement |
| 2013 | Yayama | Yes | Yes | Yes | Yes | No | No | Yes | Yes | Moderate | well described; limitations recognized (staff completion of documentation) |
| 2016 | Bowen | Yes | Yes | Yes | Yes | Yes | Not applicable | Yes | Yes | Moderate | Well described, limited number of falls |
| 2017 | Kumar | No | Yes | Yes | Yes | Yes | No | Yes | Yes | Poor | Secondary data - no date given, no data as to how participants were chosen |
| 2017 | Jansen | Yes | Yes | Yes | Yes | Yes | Yes | Yes | Yes | Good | Well described, confounding factors and variables discussed |
| 2018 | Bowen | Yes | Yes | Yes | Yes | Not applicable | Not applicable | Yes | Yes | Moderate | Models describing CI and gait quality / balance ability |
| 2019 | Okada | No | No | Yes | Yes | No | No | Yes | Yes | Poor | No inclusion / exclusion criteria; results are hard to interpret |
| 2019 | Bowen | Yes | Yes | Yes | Yes | Yes | Yes | Yes | Yes | Good | Well outlined |
| 2020 | Yang | No | No | Unclear | Yes | No | No | Yes | Yes | Poor | Not clear if patients in study had dementia, not clear how patients were recruited,  no statement of ethics |
| 2021 | Bowen | Yes | Yes | Yes | Yes | Yes | Yes | Yes | Yes | Good | Well described |
| 2021 | Bellini | No | Yes | Yes | Not applicable | Not applicable | Not applicable | Yes | Yes | Moderate | Mobility data used to build Popularity and Relational index |
| 2021 | Zhang | Yes | Yes | Yes | Not applicable | No | No | Yes | Yes | Moderate | 3 COVID subjects recovery based on activity levels |

Options: Yes, no, unclear, not applicable

Case studies

| Publication Year | Author | Were patient’s demographic characteristics clearly described? | Was the patient’s history clearly described and presented as a timeline? | Was the current clinical condition of the patient on presentation clearly described? | Were diagnostic tests or assessment methods and the results clearly described? | Was the intervention or treatment procedure clearly described? | Was the post intervention clinical condition clearly described? | Were adverse events (harms) or unanticipated events identified and described? | Does the case report provide takeaway lessons? | Rating | Rating comments |
| --- | --- | --- | --- | --- | --- | --- | --- | --- | --- | --- | --- |
| 2002 | Chan | Yes | Yes | Not applicable | Yes | Yes | Not applicable | Not applicable | Yes | Moderate | Data compared to nursing documentation - discrepancies |
| 2008 | Yamakawa | Yes | Yes | Not applicable | Yes | Yes | Yes | Yes | Yes | Good | Environmental controls for FTD patient |
| 2020 | Vahia | Yes | No | Not applicable | Yes | Yes | Not applicable | Not applicable | Yes | Moderate | Data compared to nursing documentation |
| 2021 | Au-Yeung | Yes | Yes | Yes | Yes | Yes | Not applicable | Not applicable | Yes | Moderate | How were participants identified, no ethics comments |

Options: Yes, no, unclear, not applicable
