# Supplementary material for: Real-time location systems technology in the care of older adults with cognitive impairment living in residential care: A scoping review
Source: Front Psychiatry. 2022 Nov 10;13:1038008. doi: 10.3389/fpsyt.2022.1038008 (PMC9685159; doi:10.3389/fpsyt.2022.1038008)
Supplement: Supplementary file 3 [file Table_1.docx]

Appendix I: Search Strategy

**Ovid MEDLINE: Epub Ahead of Print, In-Process & Other Non-Indexed Citations, Ovid MEDLINE® Daily and Ovid MEDLINE®** 1946-Present

| **#** | **Searches** |
| --- | --- |
| 1 | exp Dementia/ or exp Cognitive Dysfunction/ or exp Cognition Disorders/ or exp Alzheimer Disease/ or exp Frontotemporal Dementia/ or exp Dementia,  Vascular/ or exp Dementia, Multi-Infarct/ or Delirium, Dementia, Cognitive Disorders/ |
| 2 | (dement* or alzheimer*).tw,kf. |
| 3 | (lewy* adj2 bod*).tw,kf. |
| 4 | ((cogniti* or neurocogniti* or intellectual) adj2 (impair* or dysfunction* or disorder* or declin*)).tw,kf. |
| 5 | or/1-4 |
| 6 | Long-Term Care/ or Residential Facilities/ or Nursing Homes/ or Homes for the Aged/ |
| 7 | ((senior or elderly) adj living).tw,kf. |
| 8 | home? for the aged.tw,kf. |
| 9 | home? for the elderly.tw,kf. |
| 10 | ((care or rest or convalescen* or retirement or senior or elderly) adj home?).tw,kf. |
| 11 | ((aged or convalescent or institutional or long-term or residential or long term) adj care).tw,kf. |
| 12 | nursing home*.tw,kf. |
| 13 | (nursing adj3 (facility or facilities or residence? or center? or centre?)).tw,kf. |
| 14 | ("old age facility" or "old age facilities").tw,kf. |
| 15 | Old age home?.tw,kf. |
| 16 | LTC.tw,kf. |
| 17 | Longterm care.tw,kf. |
| 18 | Long term care.tw,kf. |
| 19 | ((residential or assisted living or long-term care or nursing or care or convalescent or retirement or rest) adj facilit*).tw,kf. |
| 20 | (residence? adj2 ('assisted living' or convalescen* or retire???? or 'long stay' or longstay or 'long term')).tw,kf. |
| 21 | institutionalization.tw,kf. |
| 22 | exp Geographic Information Systems/ or exp Monitoring, Physiologic/ or exp Monitoring, Ambulatory/ or exp Remote Sensing Technology/ or exp  Wireless Technology/ or Wearable Electronic Devices/ |
| 23 | ((beacon or monitoring or geolocation or location or assistive or surveillance or tracking) adj2 technolog*).tw,kf. |
| 24 | (resident? adj2 (monitor* or track*)).tw,kf. |
| 25 | ((remote or ambulatory or wireless or digital) adj2 (monitor* or sens*)).tw,kf. |
| 26 | continuous surveillance.tw,kf. |
| 27 | (RTLS or RFID).tw,kf. |
| 28 | Real time location*.tw,kf. |
| 29 | Global positioning*.tw,kf. |
| 30 | (radio frequency ident* or radio-frequency ident*).tw,kf. |
| 31 | or/6-21 |
| 32 | or/22-30 |
| 33 | 5 and 31 and 32 |
| 34 | 33 |
| 35 | limit 34 to "380 aged " [Limit not valid in Ovid MEDLINE(R),Ovid MEDLINE(R) Daily Update,Ovid MEDLINE(R) PubMed not MEDLINE,Ovid  MEDLINE(R) In-Process,Ovid MEDLINE(R) Publisher; records were retained] |
| 36 | limit 35 to english language |
| 37 | limit 36 to human |
